# Immunogenicity of unprocessed and photooxidized bovine and human osteochondral grafts in collagen-sensitive mice

**DOI:** 10.1186/1471-2474-7-32

**Published:** 2006-03-17

**Authors:** Jill S Kawalec-Carroll, Vincent J Hetherington, Douglas S Dockery, Carey Shive, Oleg S Targoni, Paul V Lehmann, Daniel Nadler, Dustin Prins

**Affiliations:** 1 Ohio College of Podiatric Medicine, 10515 Carnegie Avenue, Cleveland, Ohio, 44106, USA; 2 Cellular Technology Ltd., 10515 Carnegie Avenue, Cleveland, Ohio, 44106, USA; 3 Centerpulse Orthopedics, Ltd., Postfach 65, CH-8404 Winterthur, Switzerland

## Abstract

**Background:**

Autologous and allogeneic osteochondral grafts have been used to repair damaged or diseased cartilage. There are drawbacks to both of these methods, however. Another possible source for osteochondral grafting is photooxidized xenograft scaffolds. The purpose of this study was to evaluate the adaptive immune response to unprocessed and photooxidized xenogeneic osteochondral grafts in a collagen-sensitive mouse model.

**Methods:**

Unprocessed and photooxidized bovine and human osteochondral grafts were used. The grafts were implanted subcutaneously in collagen-sensitive DBA/1LacJ mice for four or twelve weeks. ELISPOT assays were conducted with spleen cells to evaluate the number of collagen-specific T cells that produce IL-2, IL-4, IL-5 or IFN-γ. Serum was collected and ELISA assays were performed to determine the titers of collagen-specific and total IgG, IgG1, IgG2a, or IgM antibodies. Histology was conducted on the retrieved osteochondral grafts.

**Results:**

Results indicated that, with respect to adaptive T cell immunity, the photooxidized bovine grafts, unprocessed human grafts and photooxidized human grafts did not induce a significant response to collagen. The unprocessed bovine grafts, however, were slightly more immunogenic, inducing a weak immune response. With respect to antibody production, the bovine grafts were less immunogenic than the human grafts. Bovine collagen-specific IgG antibodies were not induced by these grafts, but production of IgM after twelve weeks was observed with both the unprocessed and photooxidized bovine grafts. In contrast, photooxidized human osteochondral grafts induced IgG1 and IgG2a antibodies, while the unprocessed human grafts did not. Pre-existing human collagen-specific IgM antibodies were present in all mice, including sham-operated negative controls that did not receive an implant. Histological analysis revealed some degree of fibrous encapsulation and inflammatory infiltrations in both bovine and human implants, whether unprocessed or photooxidized.

**Conclusion:**

Both bovine and human cartilage grafts showed weak, but clear immunogenicity in the DBA/1LacJ mice, indicating that immunogenic collagen was still contained in the grafts, even after cleaning and photooxidation. The process of photooxidation is still important in osteochondral grafting, since it stabilizes the surface of the cartilage by cross-linking the collagen fibers, and allows for immediate load bearing and joint resurfacing.

## Background

Symptomatic defects in the articular cartilage and underlying bone of various joints can result from different means, including osteoarthritis, trauma and osteochondritis dissecans. One accepted means of treatment is osteochondral implantation, which has been used to treat chondral and osteochondral defects of the femoral condyle, talus, tibia, humeral capitulum and femoral head. [1-3] Osteochondral Autograft Transplantation (OATS) is a procedure that entails obtaining cylindrical osteochondral grafts from a minimally weight-bearing portion of the femoral condyles and transplanting them into an osteochondral defect on the weight-bearing surface of a joint. [2] There are concerns with using autogenous tissue for osteochondral grafting. These include performing surgery to harvest the grafts from an otherwise healthy joint [2] and donor site morbidity [2,4]. The use of OATS is ideally limited to smaller defects (1–4 cm^2^), to reduce donor site morbidity. [1,2]

Grafting with osteochondral allografts has been used to treat both small and large defects in various joints. [5-9] There is concern, however, about the possibility of transmitting viral diseases, such as human immunodeficiency virus (HIV) and hepatitis, from donor to recipient when implanting allogeneic tissue. [9,10] While the frequency of transmission of viral diseases is low, the risk is never completely removed. [10] Another concern is the potential for a host immune response to the donor tissue. [11-13] Sirlin et al. [12] have demonstrated that there is a correlation between an antibody response to osteochondral allografts and increased inflammatory reaction associated with less complete graft incorporation.

Another type of grafting material is osteochondral xenografts. The concern with xenografts is rejection of the graft by the recipient. Some work has been conducted in the area of treating xenogeneic heart and vascular tissue to make it more acceptable to the host, and this type of processing may be applicable to orthopaedics. Xenogeneic pericardial tissue, which contains collagen, has been treated by dye-mediated photooxidation in order to stabilize the material. A study by Moore et al. [14] examined bovine pericardial tissue treated by photooxidation and found that the resultant tissue was resistant to chemical and enzymatic digestion, maintained some of the physical properties of natural pericardial tissue, and was more resistant to *in vivo *degradation. Furthermore, analysis of soluble bovine collagen that was treated by photooxidation indicated crosslink formation. Subsequent studies using photooxidized bovine pericardium [15,16] and porcine heart tissue [17-20] have confirmed these findings. The photooxidized bovine and porcine tissues were also found to be less immunogenic than untreated tissue. [18,21]*In vivo *studies have suggested that the photooxidized material shows potential for use in the replacement of heart valves. [22-24] It should be noted, however, that photooxidized tissue is not currently used clinically for heart valve replacement. *In vitro *[25,26] and *in vivo *[27] studies have also indicated that photooxidation can be used to enhance the performance of vascular grafts.

Researchers have evaluated the efficacy of using photooxidation to improve the performance and acceptance of osteochondral xenografts for the repair or replacement of damaged articular cartilage. Akens et al. [28] conducted an *in vitro *study to assess the interaction between photooxidized bovine articular cartilage, untreated bovine articular cartilage and synovial membrane. The authors concluded that the photooxidized tissue may have a beneficial effect on adjacent host cartilage. It should be noted, however, that no viable chondrocytes were present in the photooxidized cartilage.

Several animal studies have been conducted on photooxidized osteochondral xenografts. [29-32] Kawalec et al. [29] found that, after twelve weeks implantation of photooxidized bovine osteochondral grafts in the patellar groove of rabbits, there was chronic inflammation and active bone remodeling within the grafts. In several instances, there was evidence of "bridging" between the host bone and the margins of the implants. The cartilage layer was intact and undamaged, and surrounded by a thin layer of fibrous tissue. This finding was also observed in a study by Nam et al., who evaluated osteochondral autograft transplantation in a rabbit model. [33] Akens et al. [30] implanted photooxidized bovine osteochondral grafts into the femoral condyles of sheep and found that, after twelve and eighteen months, there were fewer cystic lesions in photooxidized xenografts, compared to untreated xenografts and autografts. There was also evidence of fusion between the photooxidized graft and host cartilage, which was not observed with the other types of grafts. At both twelve and eighteen months, a hyaline-like cartilage matrix was observed for the photooxidized xenografts. von Rechenberg et al. [31] found that photooxidation of osteochondral xenografts implanted in the femoral condyles of sheep slowed down the rate of resorption in the subchondral bone of the grafts, due to the decreased immunogenicity of the tissue. This led to greater graft stability and improved the survival of the cartilage layer. In another study conducted by von Rechenberg et al. [32], the shape of the photooxidized osteochondral graft was found to be important. Mushroom-shaped grafts were associated with fewer cystic lesions, less fibrous tissue, and more complete bone remodeling, compared to cylindrical grafts. As in the previous study, the stability of the subchondral bone was significant in the survival of the cartilage layer.

In order to further evaluate the immune response to photooxidized osteochondral xenografts, Hetherington et al. [34] evaluated the innate immunological response in humans towards osteochondral xenografts. Healthy peripheral blood mononuclear cells were challenged with antigens of bovine, porcine, ovine and equine origin that had been partially or entirely treated by the photooxidation process. Untreated bovine, porcine and equine specimens resulted in a vigorous cytokine response. After the first step in processing, which involved cleaning the specimens in increasing strengths of ethanol, there was no significant activation of cells of the innate immune system. This lack of response continued through successive steps of the process, including photooxidation. It was concluded that processing of the osteochondral grafts dramatically, if not completely, negated the immunostimulatory properties of the test sample.

The long-term goal of this current research is to evaluate the possibility of using photooxidized xeno-derived osteochondral grafts as a repair matrix for damaged or diseased cartilage. The graft is subjected to photooxidative treatment because the process preserves the mechanical integrity of the cartilage by cross-linking the collagen fibers. It is presently unknown how this treatment affects the acquired immunogenicity of the graft and hence its long-term immune acceptance by the recipient. The purpose of this study was to evaluate the adaptive cellular and humoral immunologic response to unprocessed and photooxidized bovine and human osteochondral grafts in an *in vivo *murine animal model.

## Methods

### Osteochondral grafts

The grafts used in this study were cylindrical plugs, approximately 3.8 mm in diameter and 5 mm in length. Each graft consisted of either bovine or human cartilage with underlying bone. The bovine grafts used in the study were obtained from the shoulder joint of the animal. They were tested as either unprocessed (Bo-UP) or photooxidized (Bo-P). The human grafts were obtained from a bone bank and were taken from a cadaveric knee joint. They were examined as either unprocessed (Hu-UP) or photooxidized (Hu-P).

### Photooxidation

After harvesting, the grafts were cleaned and rinsed thoroughly, to remove bone debris and marrow fat. Designated grafts were then subjected to the photooxidation process. [14,19,30-32] Briefly, the grafts were submerged in a 0.01% methylene blue solution in phosphate-buffered saline (PBS) while exposed to a light source under controlled conditions (light intensity, temperature, and exposure time). After exposure, the photooxidized grafts were rinsed and cleansed thoroughly in PBS to remove excess methylene blue.

### Experimental model

The animal model chosen for this study was the collagen-sensitive DBA/1LacJ mouse (Jackson Laboratory, Bar Harbor, ME, USA). Grafts were implanted subcutaneously for either four or twelve weeks. Each of the experimental mice was first anesthetized with an anesthesia cocktail, and the dorsum was shaved and swabbed with Betadine. A small incision was created and an implant was inserted subcutaneously. The incision was then closed with nylon suture. In addition to the experimental mice, several mice were given a sham operation to serve as a negative control. As additional controls, groups of mice were challenged with bovine collagen as shown in Table 1, while others were challenged with human collagen, as shown in Table 2. The number of animals per group is also shown in Tables 1 and 2. The mice were cared for in accordance with the guidelines of the OCPM Institutional Animal Care and Use Committee.

At four or twelve weeks, blood was obtained from the tail vein of mice from each group. The mice were then euthanized with CO_2 _and the spleen cells were isolated for T cell assessment.

### Positive controls

Positive controls were established in this study, using purified bovine type II collagen or human collagen (Chondrex, Redmond, WA, USA) administered with incomplete Freund's adjuvant (IFA) (Gibco BRL, Grand Island, NY, USA) to induce a type 2 immune response. Another set of mice received bovine or human collagen administered with complete Freund's adjuvant (CFA) to induce a type 1 immune response. The CFA was produced by mixing inactivated *M. tuberculosis *H37RA (Difco Laboratories, Detroit, MI, USA) at 1 mg/ml into IFA. The antigens were mixed with adjuvants to a final concentration of 1 mg/ml. A total of 400 μl of antigen in either IFA or CFA was injected intraperitoneally (IFA) or subcutaneously (CFA). All positive controls were euthanized with CO_2 _three weeks after injection, and tested as described below.

### ELISPOT assays

The frequency of collagen -specific T cells in the spleen and the Th1/Th2 effector lineage of these cells were determined using cytokine ELISPOT by measuring collagen-induced IFN-γ, IL-2, IL-4 and IL-5 production. ImmunoSpot^® ^M200 plates (Cellular Technology Ltd., Cleveland, OH) were coated overnight at 4°C with the different cytokine-specific capture antibodies specified below. The plates were then blocked with 1% bovine serum albumin (BSA) in PBS for one hour at room temperature, and washed four times with PBS. Subsequently, freshly isolated spleens cells were plated at 10^6 ^cells per well, in two to four replicate wells with or without the nominal antigen or control antigens. The assay medium was serum-free HL-1 (Biowhittaker, Walkersville, MD, USA) supplemented with 1 mM L-glutamine.

Following 24 hours (for IFN-γ and IL-2 measurements) or 48 hours (IL-4 and IL-5 measurements) of cell culture in the incubator at 37°C, the cells were removed by washing three times with PBS and then four times with PBS containing 0.05% Tween-20 (PBST). The different biotinylated detection antibodies specified below were added. After overnight incubation at 4°C, the plates were washed three times with PBST followed by incubation for two hours at room temperature with streptavidin-ALPH conjugate (Dako Corp., Carpenteria, CA, USA) at 1:1000 dilution. Plates were then washed two times with PBST and twice with PBS. The NBT/BCIP substrate (50% NBT buffer, 33% stock NBT, and 17% stock BCIP) was added and left for 15–30 minutes. The reaction was stopped by rinsing with tap water, and the plates were allowed to dry overnight. The following coating antibodies were used for IL-2, IL-4, IL-5 and IFN-γ, respectively: JES6–1A12 (2 μg/ml), BVD4-1D11 (4 μg/ml), TRFK5 (5 μg/ml) and R46A2 (2 μg/ml). The detection antibody concentrations were as follows: JES6-5H4-biotin (1 μg/ml), BVD4-24G2-biotin (1 μg/ml), TRFK4-biotin (1 μg/ml) and XMG1.2-biotin (2 μg/ml). All antibodies were purchased from BD-Pharmingen, San Diego, CA.

### Computer-assisted ELISPOT image analysis

The image analysis was performed using a Series 3B ImmunoSpot^®^Image Analyzer (Cellular Technology, Ltd.). Digitized images were analyzed for the presence of areas in which color density exceeded background by a factor set on the basis of the comparison of control wells (containing T cells and antigen-presenting cells (APC) without antigen) and experimental wells containing collagen as the antigen. After separating spots that touch or partially overlap, additional criteria of spot size and circularity were applied to gate out speckles and noise caused by spontaneous substrate precipitation, nonspecific antibody binding, and "ELISA effects". Objects that did not meet these criteria were ignored and areas that met them were recognized as spots, counted, and highlighted.

### Measurement of specific serum antibodies

Standard ELISA assays were conducted to determine a humoral-mediated immune response. These assays measured the titers of collagen-specific antibodies in the sera of the mice, specifically IgG1, IgG2a, total IgG and IgM. After blood was obtained from the tail vein of the mouse, the cells were pelleted by centrifugation and the serum was collected. Plates (Nunc Immunoplate, Fisher Scientific, Pittsburgh, PA, USA) were coated with bovine type II collagen (10 μg/ml) or human collagen (5 μg/ml) diluted in bicarbonate buffer overnight at 4°C, then blocked for one to two hours with 0.1% gelatin in PBST. The test serum was added and incubated overnight at 4°C. Plate-bound antibody was detected by biotinylated anti-mouse immunoglobulin. Rat anti-mouse IgG from Zymed (San Francisco, CA, USA) was used to detect total Ig; the isotype-specific antibodies used to detect IgG1, IgG2a, and IgM were also purchased from Zymed. Tertiary antibody, streptavidin-alkaline phosphatase was purchased from Dako (Carpenteria, CA, USA) and p-Nierophenyl phosphate disodium salt (PNPP) was used for the development of the colorimetric reaction.

### Histological analysis

After removal from the implantation site, each graft was placed in 10% buffered formalin solution for a minimum of seven days. The retrieved specimens were then decalcified, embedded in paraffin, sectioned and stained with hematoxylin and eosin. All sections were reviewed by a blinded pathologist.

### Statistical analysis

Statistical analysis was conducted on the cytokine results. Tests used to determine statistical significance included the two-tailed student's t test and the Mann Whitney Rank Sum test. In both instances, significance was defined as p < 0.05.

## Results

### Bovine grafts

**Figure 1:**
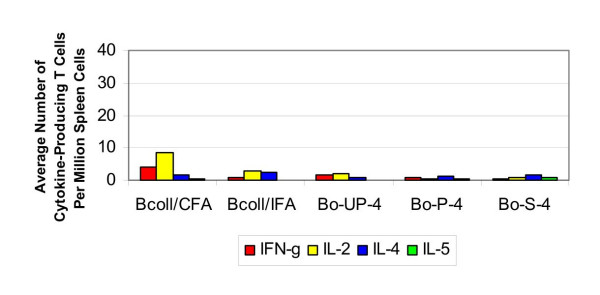
**Cytokine response to bovine collagen (4 weeks). **The average number of cytokine-producing T cells per million spleen cells in response to a challenge from bovine collagen and osteochondral grafts at four weeks.

The average number of collagen-specific cytokine-producing T cells per million spleen cells at four weeks post-implantation are shown in Figure 1. In CFA:collagen-immunized positive control mice, the IFN-γ and IL-2 responses were significantly greater than those engaged in mice receiving photooxidized grafts after four weeks (p < 0.05). The CFA:collagen immunization generated a significantly greater IL-2 response compared to the unprocessed bovine graft. Regarding the IFA:collagen-immunized positive controls, the cytokine response to the positive controls was not significantly different than that induced by either the unprocessed or photooxidized grafts. The presence of neither the unprocessed nor the photooxidized grafts induced a significant increase in cytokine production versus sham mice. The IL-2 response to photooxidized grafts was significantly less than the same response to the unprocessed grafts. There was no significant production of IL-4 or IL-5 by any mice.

ELISA testing indicated that, after four weeks, bovine collagen-specific antibodies were not induced in the sham-operated mice. There was no elevation of bovine collagen-specific antibodies in response to either unprocessed or photooxidized bovine osteochondral grafts.

**Figure 2:**
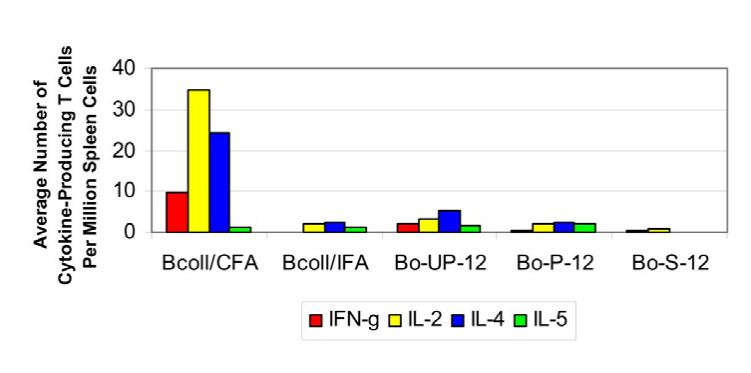
**Cytokine response to bovine collagen (12 weeks). **The average number of cytokine-producing T cells per million spleen cells in response to a challenge from bovine collagen and osteochondral grafts at twelve weeks.

The number of collagen-specific cytokine-producing T cells per million spleen cells at twelve weeks post-implantation in mice that received bovine osteochondral grafts are shown in Figure 2. Collagen-specific T cells were present in significantly higher frequencies in the CFA:collagen-immunized positive controls than in mice that have received unprocessed and photooxidized grafts (p < 0.05). There was no significant difference between the cytokine responses to collagen in the IFA:collagen-immunized control mice and the mice that received photooxidized grafts. The IFN-γ response to the unprocessed grafts, however, was significantly greater than the response in the IFA:collagen-immunized controls. The photooxidized grafts did not induce a significant T cell response to collagen over what was seen in sham treated control mice, while the unprocessed graft produced an elevated IL-4 response versus the negative controls. The IFN-γ response to the photooxidized grafts was significantly less than the response to the unprocessed grafts. There was no significant IL-5 elevation in any of the mice.

ELISA testing showed that twelve weeks after the treatment bovine collagen-specific IgG and IgM antibodies were not produced in the sham-operated mice. The unprocessed bovine grafts did not induce production of bovine collagen-specific IgG, IgG1 and IgG2a antibodies. Specific IgM antibodies, however, were produced in response to the unprocessed material. Similarly, only bovine collagen-specific IgM antibodies were produced after twelve weeks exposure to photooxidized grafts.

### Human grafts

**Figure 3:**
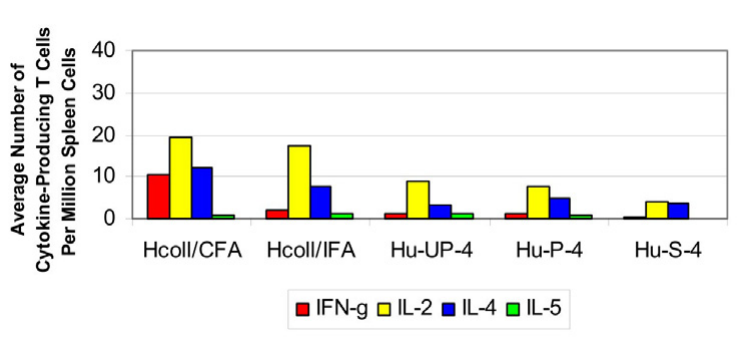
**Cytokine response to human collagen (4 weeks). **The average number of cytokine-producing T cells per million spleen cells in response to a challenge from human collagen and osteochondral grafts at four weeks.

The average number of collagen-specific cytokine-producing T cells per million spleen cells at four weeks post-implantation in mice that received human osteochondral grafts is shown in Figure 3. The IL-4 response induced by CFA:collagen immunization was significantly greater than that induced by either the unprocessed or photooxidized grafts (p < 0.05). In addition, the IFN-γ response induced by the unprocessed grafts was significantly less than that induced in response to the CFA:collagen-immunized positive controls In IFA:collagen-immunized controls, the collagen-induced IL-2 production was significantly greater than that induced by the photooxidized grafts. Also the collagen-induced IL-4 production in the IFA:collagen-immunized control mice was significantly greater than that in the mice that received unprocessed grafts. Neither the unprocessed nor photooxidized grafts induced a significant T cell response versus the sham mice. There was no significant difference between the cytokine responses induced by the unprocessed and photooxidized grafts. Also, there was no significant IL-5 elevation in any of the mice.

ELISA testing showed, after four weeks, production of human collagen-specific IgG antibodies was not induced in the sham-operated negative control mice, however, these mice demonstrated elevated levels of IgM antibodies. Similarly, there was not an increased production of human collagen-specific IgG antibodies in response to unprocessed human grafts, but there was an elevated specific IgM response. In response to the photooxidized human grafts, there was no elevated human collagen-specific total IgG and IgG2a production, while a weak IgG1 response was observed in two of eight mice. There was elevated IgM production in response to the photooxidized grafts.

**Figure 4:**
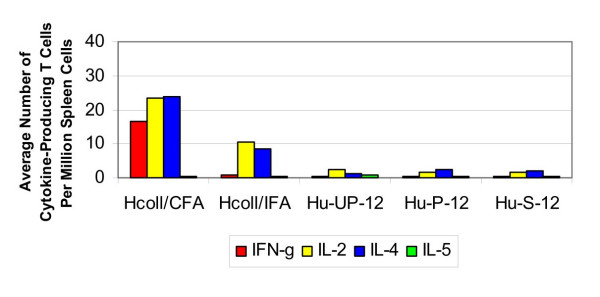
**Cytokine response to human collagen (12 weeks). **The average number of cytokine-producing T cells per million spleen cells in response to a challenge from human collagen and osteochondral grafts at twelve weeks.

The average number of collagen-specific cytokine-producing T cells per million spleen cells at twelve weeks post-implantation in mice that received human osteochondral grafts are shown in Figure 4. The numbers of collagen-specific IFN-γ, IL-2 and IL-4 producing T cells were significantly higher in CFA:collagen-immunized mice than in mice that received unprocessed or photooxidized grafts (p < 0.05). IL-2 and IL-4 production was also significantly greater for the IFA:collagen-immunized control mice, compared to mice receiving either an unprocessed or a photooxidized graft. The T cell response to the unprocessed and photooxidized grafts was not significantly different than that generated by the negative control mice. There was no significant difference between the cytokine response induced by the unprocessed grafts and that induced by the photooxidized grafts. IL-5 production was negligible in all mice.

ELISA testing showed that, after twelve weeks, there was no increased production of human collagen-specific IgG antibodies in sham-operated mice, but there were elevated IgM titers in these mice. There were no elevated serum levels of collagen-specific IgG antibodies in mice grafted with unprocessed human cartilage, while IgM titers were high in these mice. With respect to the photooxidized human grafts, two of the seven mice showed high specific IgG1 titers, and one additional mouse showed an elevated IgG1 level. These grafts also induced high titers of specific IgG2a antibodies in one mouse, and moderate levels of IgG2a in another mouse. There were elevated titers of collagen-specific IgM in all mice implanted with the photooxidized graft.

### Histological findings

The histological findings were similar for bovine and human grafts, regardless of whether the grafts were unprocessed or photooxidized. All grafts showed some degree of fibrous encapsulation. The degree and extent of encapsulation varied and showed no consistent trend related to graft type, processing or duration of implantation. The cartilage and bone revealed the absence of chondrocytes and osteocytes, respectively. All grafts demonstrated some degree of inflammation ranging from mild to moderate and can best be described as mixed in character, with evidence of both acute and chronic inflammatory processes. The inflammation was observed to begin at the periphery of the implants at four weeks, with extension of an inflammatory or fibroinflammatory infiltrate into the medullary spaces at twelve weeks.

## Discussion

This study was conducted to assess the immunogenicity of osteochondral tissue grafts. The collagen-sensitive murine model used in this study was selected along with a subcutaneous site for implantation to ensure a "worst case scenario" situation. The grafts consist of bone and cartilage, and collagen is a main protein constituent of the implanted material. When immunized with type II collagen, the DBA/1LacJ mice develop a strong immune response to collagen which results in severe autoimmune arthritis. [35] In this current experimental system, such immunizations with collagen were used as the positive control, measuring the magnitude of the ensuing T- and B- cell responses. Subcutaneous antigen encounter favors the induction of an immune response because of the high density of dendritic cells in the skin – skin grafts therefore can be expected to be more immunogenic than joint implants.

The frequencies of collagen-specific T cells as identified by their cytokine signature in ELISPOT assays showed that the implantation of bovine photooxidized osteochondral grafts did not induce detectable T cell immunity within four or twelve weeks. While no response was detected in mice that received the unprocessed graft after four weeks, a slight elevation in IL-4 after twelve weeks in comparison to the sham-operated mice suggests a possible weak cellular Th2 response in these mice. In certain instances at both time periods, the unprocessed grafts were moderately more active compared to the photooxidized grafts. The fact that none of the mice, including the positive controls, elicited an elevated IL-5 response after twelve weeks suggests that this arm of the Th2 response may not be participating in the response to bovine collagen.

Bovine collagen-specific total IgG, IgG1 and IgG2a antibodies were not induced by the unprocessed or photooxidized bovine osteochondral grafts at either four or twelve weeks. By twelve weeks, however, specific IgM antibodies emerged in all of the mice receiving either type of graft. IgM antibodies did not appear at either time period in the mice receiving a sham operation, thus suggesting that mice that received the bovine grafts displayed a weak, delayed IgM antibody response. The production of IgM antibodies does not require T cell help. The fact that these collagen-specific IgM antibodies appeared late, and in the absence of T-cell dependent IgG antibodies, along with the lack of detectable T cell activity suggest such a T cell independent weak IgM B cell response.

Measurements of collagen-induced cytokine production in mice implanted with human osteochondral grafts confirmed that neither the unprocessed nor the photooxidized samples induced a significant T cell response after four or twelve weeks. This would suggest that, with respect to adaptive T cell immunity, either implant type would be suitable for transplantation.

None of the mice grafted with unprocessed human osteochondral grafts generated human collagen-specific total IgG, IgG1 or IgG2a antibodies at either time period. However, a weak IgG1 response was generated by four weeks after implantation with the photooxidized human graft, with a further increase in IgG1 levels at twelve weeks. In addition, one mouse exhibited high IgG2a production after twelve weeks. These antibodies are dependent on antigen-specific T cell help suggesting that T cell immunity was induced. While IgG1 antibodies are present in Th1 and Th2 immunity, B cell isotype switching to IgG2a requires IFN-γ and thus is linked to Th1 immunity. Strikingly, these mice showed "natural IgM" antibodies to collagen, that is, even untreated "naïve" mice had collagen-specific IgM antibodies in their serum. Such "natural antibodies" are typically induced by environmental stimuli, such as the bacterial flora; the blood group antibodies being the best known examples. It is likely that these "natural" anti-human collagen antibodies in mice are directed against glycosylation differences between the two species.

The appearance of graft-specific antibodies clearly shows that the indirect pathway of graft rejection has been engaged. This mechanism, first introduced by Benichou et al. [36], entails the sensitization of recipient T cells to processed antigens of donor origin that are expressed on the surface of recipient antigen-presenting cells. Accompanying the T cell response, a B cell response can also be engaged, resulting in the production of antibodies directed against the graft. [37] The indirect pathway can result in graft rejection even in the absence of the classical direct pathway (in which T cells react when exposed to allo- or xeno-peptide complexes that are expressed on the surface of donor antigen-presenting cells), however, rejection resulting from the indirect pathway is typically prolonged. [38] As with the induction of any T cell response, it is required that the antigen-presenting cells of the innate immune system are in an activated state in order to prime the T cells that mediate indirect graft rejection. The processed allo- or xeno-antigen can then be linked with enhanced costimulation, resulting in rejection. In the absence of such activation-dependent costimulation, tolerance develops. By measuring the activation of the cells of the innate immune system, information on the engagement of costimulation can be provided.

The results from this study and a previous study conducted by Hetherington et al. [34] provide an overall analysis of both the innate and the adaptive immune responses to photooxidized osteochondral xenografts. The findings are summarized in Table 3. As can be seen, there is a considerable reduction in the innate immune response after processing the osteochondral grafts. While the adaptive T cell response is negligible in both unprocessed and photooxidized implants, the humoral response to both graft types needs to be addressed to determine why a response, particularly IgM, is induced.

The histologic appearance of the explanted grafts, which showed fibrous encapsulation and infiltration of acute and chronic inflammatory components into the graft itself, is inconsistent with results seen in previous works, in which the grafts were inserted into bone as opposed to subcutaneous implantation in this study. [29-32] A previous study conducted by three of the authors (JSK-C, VJH and DN) in which osteochondral xenografts were implanted into the patellar groove of rabbits demonstrated a nonspecific inflammatory response with the implants being well tolerated and, in some instances, the graft bone was in the initial stages of incorporation into the host bone. [29] The inflammation observed in this study appeared to be more intense and may be accounted for by a difference in implantation site, with the subcutaneous location favoring immune sensitization of the host. The use of a collagen-sensitive mouse may also affect the response to the graft itself. To clarify the mechanism underlying the inflammatory response, two of the authors (JSK-C and VJH) implanted two photooxidized bovine osteochondral grafts subcutaneously into immunodeficient Rag-1 gene knock out mice (B6.129S7-*Rag1*^*tm*1*Mom*^/J, Jackson Laboratory, Bar Harbor, ME, USA). These mice are deficient in mature B cells or T cells. The mice were sacrificed four weeks after grafting and the implants were subjected to histological analysis. The inflammatory reaction to the grafts seen in these immunodeficient mice was similar in quality and magnitude to that of the collagen-sensitive mice. This outcome suggests that the inflammatory response observed in the grafts results from a response of the innate immune system and that the IgM antibodies seen in the immune competent mice did not play a detectable role in intensifying it.

It should be noted that the grafts were tested in the same form as would be used clinically. The cylindrical graft consisted of a cartilage layer with underlying bone. However, the location of implantation was not clinically relevant. As indicated above, the subcutaneous implantation site was chosen in order to achieve a stronger immunological response. Previous studies have also indicated that, histologically, photooxidized grafts are better accepted by the host, both in non-weight-bearing [29] and weight-bearing [30-32] areas of the joint. The next logical step in this current research is to evaluate the immune response to the photooxidized implant when placed in a clinically-relevant weight-bearing joint.

## Conclusion

Both bovine and human cartilage grafts showed weak, but clear immunogenicity in the DBA/1LacJ mice. Regarding T cell immunity, the unprocessed bovine grafts were slightly more immunogenic than photooxidized bovine grafts, unprocessed human grafts and photooxidized human grafts. With respect to antibodies, bovine grafts were less immunogenic than human grafts, but both photooxidized and unprocessed bovine grafts induced a late IgM response. Bovine collagen-specific IgG antibodies were not induced by these grafts. In contrast, photooxidized but not the unprocessed human cartilage grafts induced IgG1 and IgG2a antibodies.

In conclusion, the data clearly show that immunogenic material is still contained in the graft, and the adaptive immunogenicity is not reduced by photooxidation, as suggested by the emergence of antibodies, primarily IgM. Photooxidation of the osteochondral grafts is still important, however, in order to retain the mechanical integrity of the cartilage, thus allowing for immediate joint resurfacing. The next phase of the study is to evaluate the immune reactions when the graft is placed into a weight-bearing joint. In addition, evaluation of the photooxidized material for the presence of viral or other residual infectious materials, such as α-galactase, should be conducted. It may also be helpful to evaluate the innate immune response to human photooxidized osteochondral grafts, using peripheral blood mononuclear cells from healthy human donors, as human material was not tested in the immunological analysis of the innate immune response conducted previously by the authors. [34]

## Competing interests

The research project was funded by Centerpulse Orthopedics Ltd. It is not known whether they will gain financially from the publication of this paper.

## Authors' contributions

JSK-C participated in the design of the study, coordinated the animal surgeries, was responsible for animal care, coordinated the collection of blood and spleen cells, assisted with data analysis, and drafted the manuscript. VJH helped conceive the study, participated in its design and coordination, assisted with animal surgeries and assisted with analysis of findings. DSD performed animal surgeries, assisted with animal care, and assisted with data analysis. CS performed injections for positive controls, collected blood and spleen cells, performed the ELISA and ELISPOT assays, assisted with data analysis and helped to draft the manuscript. OST assisted with harvesting the spleens and with the ELISA and ELISPOT assays. PVL participated in the design of the study and assisted with data analysis. DN participated in the design of the study and prepared the osteochondral grafts. DP assisted with data analysis. All authors read and approved the final manuscript.

## Pre-publication history

The pre-publication history for this paper can be accessed here:



## Figures and Tables

**Table 1:** Bovine experimental groups

BColl/CFA (n = 4 for comparison to 4-week groups) (n = 4 for comparison to 12-week groups)	Positive control; bovine collagen with complete Freund's adjuvant, euthanized 3 weeks after injection
BColl/IFA (n = 4 for comparison to 4-week groups) (n = 4 for comparison to 12-week groups)	Positive control; bovine collagen with incomplete Freund's adjuvant, euthanized 3 weeks after injection
Bo-UP-4 (n = 7)	Unprocessed bovine graft, 4 weeks implantation
Bo-P-4 (n = 8)	Photooxidized bovine graft, 4 weeks implantation
Bo-S-4 (n = 4)	Negative control; sham operation, euthanized 4 weeks post-op
Bo-UP-12 (n = 8)	Unprocessed bovine graft, 12 weeks implantation
Bo-P-12 (n = 8)	Photooxidized bovine graft, 12 weeks implantation
Bo-S-12 (n = 4)	Negative control; sham operation, euthanized 12 weeks post-op

B or Bo = bovine; UP = unprocessed; P = photooxidized; S = sham

**Table 2:** Human experimental groups

HColl/CFA (n = 8 for comparison to 4-week groups) (n = 4 for comparison to 12-week groups)	Positive control; human collagen with complete Freund's adjuvant, euthanized 3 weeks after injection
HColl/IFA (n = 8 for comparison to 4-week groups) (n = 4 for comparison to 12-week groups)	Positive control; human collagen with incomplete Freund's adjuvant, euthanized 3 weeks after injection
Hu-UP-4 (n = 8)	Unprocessed human graft, 4 weeks implantation
Hu-P-4 (n = 8)	Photooxidized human graft, 4 weeks implantation
Hu-S-4 (n = 4)	Negative control; sham operation, euthanized 4 weeks post-op
Hu-UP-12 (n = 8)	Unprocessed human graft, 12 weeks implantation
Hu-P-12 (n = 7)	Photooxidized human graft, 12 weeks implantation
Hu-S-12 (n = 8)	Negative control; sham operation, euthanized 12 weeks post-op

H or Hu = human; UP = unprocessed; P = photooxidized; S = sham

**Table 3:** Summary of immunology studies on photooxidized osteochondral xenografts

	Unprocessed	Photooxidized
Macrophages, cells of the innate immune system (ref. 34)	++++++	+
Adaptive T cell response to bovine grafts (cellular)	+	-
Adaptive T cell response to human grafts (cellular)	-	-
Adaptive B cell response to bovine grafts (humoral) – IgG1, IgG2a, IgG	-	-
Adaptive B cell response to human grafts (humoral) – IgG1, IgG2a, IgG	-	+
Adaptive B cell response to bovine and human grafts (humoral) – IgM	++	++
